# Using search engine analytics to identify public interest and needs in periodontal disease: a retrospective comparative search term analysis of the keywords “periodontitis” and “gingivitis” for the years 2008–2025 in 5 different countries

**DOI:** 10.3389/fdgth.2026.1746444

**Published:** 2026-02-04

**Authors:** Pablo Cores Ziskoven, Christian Walter, Philipp Bani, Lena K. Müller-Heupt, Andreas M. Geyer, David Kiramira, Dominik Haag, James Deschner

**Affiliations:** 1 Department of Periodontology and Operative Dentistry, University Medical Center, Johannes Gutenberg University, Mainz, Germany; 2 Oral and Maxillofacial Surgery Mediplus MVZ GmbH, Mainz, Germany; 3 Department of Oral and Maxillofacial Surgery, Plastic Surgery, University Medical Center, Mainz, Germany

**Keywords:** digital health behavior, gingivitis, googletrends, periodontitis, personalized medicine, search term analysis

## Abstract

**Background:**

Periodontitis is a widespread disease with high global prevalence. Alongside medical consultations, patients and healthcare professionals increasingly seek diagnostic and treatment information online. However, few studies have analyzed these search queries, despite their potential to reflect public interest and concerns. Extracting and analyzing such queries may contribute significantly to personalized diagnostics, therapy, and prevention, while also raising awareness of the disease.

**Methods:**

The web tool *Google Trends* and its extension *Glimpse* were used to identify and analyze the relative and absolute search volumes of “periodontitis” and “gingivitis”, along with related queries, in five countries (Germany, Italy, France, Spain, and the USA) for the years 2008–2025. Additionally, *Ubersuggest* provided data on search volumes, device types, age groups, and click behavior. Statistical analysis was conducted using *GraphPad Prism Software* and included linear regression, distribution analysis and comparison as well as Chi-squared tests.

**Results:**

From 2008 to 2025, search volumes for both terms increased significantly in all five countries. In Germany and Italy, “periodontitis” overtook “gingivitis”, while in France, Spain, and the US, “gingivitis” remained more frequently searched. Age-specific trends showed gingivitis queries peaked among 18–34-year-olds, while periodontitis was most searched by those aged 25–44. Most searches were conducted via mobile devices, especially in Italy and the USA. Around 50% of users did not click on any result, and organic results were preferred over advertisements. Related queries highlighted strong interest in treatment, home remedies, veterinary relevance, and—uniquely in Spain—celebrity connections.

**Conclusion:**

Search interest in gingivitis and periodontitis is high and has grown significantly from 2008 to 2025 across all examined countries. This suggests increased awareness but also reveals a need for improved education and accessible information. Frequently searched topics focused on etiology, diagnosis, and treatment, underscoring the role of search engines as key sources of medical information. These insights offer valuable, unfiltered reflections of patient concerns and questions. In an era of personalized medicine, such data should be integrated into patient-centered care strategies to better address individual needs.

## Introduction

Periodontitis is a biofilm-associated chronic inflammatory disease of the periodontium, which can lead to host-induced attachment loss and thus to tooth loosening and tooth loss (1). Periodontitis itself, but also its consequences, are associated with considerable restrictions in quality of life and a shortened lifespan, as (premature) tooth loss can have a negative impact on food intake, phonetics, aesthetics, etc., but also on mental health (2–4). Furthermore, it has been shown that periodontitis as well as tooth loss is partly bidirectionally associated with general diseases such as diabetes, coronary heart disease, etc. and overall higher all-cause mortality (5–8). The latest German wide national epidemiological studies (DMSVI) have shown that around 95% of younger adults and 85% of senior citizens in Germany are affected by periodontitis, with the severity increasing with age (9). As recent studies have shown, periodontitis is also one of the most common diseases in the population worldwide and is a widespread disease that also has social consequences, as its late treatment causes avoidable high costs for the health system and economy (10, 11). Furthermore, due to demographic changes, a growing number of patients—particularly aging populations who increasingly include digital natives—will be affected and are expected to seek health information online. The urgency of tackling this disease comprehensively has been emphasized by recent calls for global action (12, 13). Those affected often notice the disease very late, as it progresses for a long time with only mild or no noticeable symptoms such as pain. Clinical symptoms such as tooth loosening or pain usually occur in the final stages of the disease, which makes treatment and follow-up care more complex and cost-intensive and worsens the prognosis. In the vast majority of cases, the progression of the disease can be prevented by early detection and preventive measures, as the treatment of an early stage of periodontitis is fairly low invasive and provides predictable results (14).

Compared to recent decades, the internet is an easily accessible and low-threshold source of medical information for the vast majority of people, in addition to conventional visits to the doctor (15). It has been shown that search engines and social networks are frequently used and even preferred (16–19) sources to obtain health data, as this way of searching is less cost-intensive, interactive and anonymous (20). Search engines are therefore an important supplement (not a substitute) for conventional visits to the doctor. Over the past decades, the proportion of individuals using the internet to obtain health-related information has steadily risen across EU Member States. According to Eurostat, by April 2025 approximately 52% of people in the European Union (aged 16–74) used the internet to seek health information, up from 34% in 2010. In 2024 and early 2025, in countries like Finland, the Netherlands, Denmark, and Germany, this figure exceeded 70%, reaching about 79% in Finland, 78% in the Netherlands, 72% in Denmark, and 57% in Germany. In the United States, a similar upward trend is observed: an estimated 61% of adults cited health information as their first online search in 2008, rising to approximately 74% by 2017 (21). In various Asian regions, the share is even higher, ranging from 79% in mainland China, 80% in the Philippines, 85% in Hong Kong, 85% in Indonesia, to 86% in Vietnam (17, 22). This offers immense potential for educational work in the periodontal field and, for example by providing self-diagnostic tools or launching awareness campaigns to increase visibility and awareness of the disease. On the other hand, the analysis of search terms and questions entered into search engines provides an unfiltered insight into the interests and needs of the population and those affected. Especially in the age of personalized medicine, this offers the opportunity to address these questions specifically in order to provide patients with the support they need. The interest and requests for information should be adequately served and directed by the relevant professional associations, among others, in order to provide patients with high quality, reliability and easy access to these health topics.

Search term analyses have been used successfully for many years in the media and advertising sector for commercial purposes in order to identify the interest of population groups in certain topics and, for example, to place personalized advertising (23). The term “search volume” refers to the absolute number of searches for a specific search term within a defined period of time. It is also possible to extract topics or questions that were also entered by searchers. This allows conclusions to be drawn about people's interests and needs and how people use the internet privately to access health information. However, these methods can also be used for non-commercial scientific purposes to draw conclusions about the “awareness” of the disease in the population and, in particular, to address the questions that are frequently searched for.

There is a consensus that periodontitis is a highly prevalent disease that can have drastic consequences for the individual patient as well as being a major burden on society (10, 13). While the epidemiology, clinical consequences, and socioeconomic burden of periodontal diseases are well studied, considerably less is known about how populations actively seek information on these conditions in digital environments. In particular, long-term, population-level analyses of online search behavior related to gingivitis and periodontitis remain scarce. Digital search analytics offer a novel opportunity to complement classical epidemiological data by capturing unfiltered, real-world information needs, temporal dynamics, and cross-national differences in health-related awareness. While a limited number of studies have applied Google Trends analyses in dentistry and oral health research, these have mainly focused on other oral conditions such as oral cancer or dental pain. To the best of our knowledge, no previous study has systematically examined long-term digital search behavior related to gingivitis and periodontitis across multiple countries over an extended time frame. By applying search engine analytics over a 17-year period and comparing five countries with different healthcare systems, this study aims to provide new insights into public interest, awareness, and unmet informational needs related to periodontal diseases. In the context of digital epidemiology and personalized medicine analyzing the search terms and questions of the population could help to increase awareness, provide help for self-help and respond to individual needs in order to reduce the burden of periodontitis on both individuals and society as a whole.

## Materials and methods

The “Google Trends” software with the “Glimpse” extension was used to analyze the digital search behaviour and search volumes in 5 countries (Germany, Italy, France, Spain and the USA). This software is generally used commercially by advertisers to analyze the search behavior of potential buyers of products and to identify related topics in order to carry out so-called “search engine optimization” (SEO). These are measures designed to improve the ranking of a website in the search results of search engines such as Google. The aim is to make websites more visible for certain search terms (keywords) in order to attract more visitors. As this technology indicates both the monthly search volume estimated by Google and the absolute search volumes of recent years, it can also be used for scientific questions.

In our study, the monthly search volumes for the keywords “periodontitis” and “gingivitis” and its translation into the corresponding language (https://www.Deepl.com) were collected for each country, analyzed and compared with each other. The analysis was limited to the defined keywords “gingivitis” and “periodontitis” and their language-specific translations; common lay synonyms, duplicate terms, spelling variations, language-specific colloquial expressions, and multi-word queries were not systematically included. Furthermore, search terms related to gingivitis and periodontitis were determined. The “Ubersuggest” website works in a similar way but allows a more in-depth analysis of the type of device used, the age of the searcher and the click behavior. Both tools use the application programming interface (API) and data from Google. As Google's market share in the studied countries is around 90% the focus on this searchterm analysis focused on Google (24). The included countries were chosen to represent different Western healthcare systems with high and comparable internet access, reliable Google market dominance, and sufficient longitudinal data availability. The USA was included as a non-European comparator with a distinct healthcare structure and digital health behavior. Google Trends is freely accessible, whereas Glimpse and Ubersuggest provide limited free access with additional features available via paid subscriptions.

### Statistical analysis

The statistical analysis was performed using the software GraphPad Prism [GraphPad 10.4.1 Software, San Diego, CA, USA, (RRID:SCR_002798)]. To assess the temporal trends in search interest, linear regression was conducted for the term “periodontitis” and “gingivitis” for each country. To determine whether the average search volumes for the terms periodontitis and gingivitis differed significantly within and between each country, independent two-sample t-tests were performed. For each country, the mean monthly search volumes across the study period (2008–2025) were compared between the two terms. Prior to testing, data were checked for completeness and normal distribution. If non-normal distribution was identified by Shapiro–Wilk test Mann–Whitney-*U*-Test was performed. Missing values were excluded pairwise. Statistical significance was defined as *p* < 0.05. Statistical analyses were also conducted to evaluate differences in search behavior across devices, age groups, and countries, as well as between the terms periodontitis and gingivitis. Age distribution data, presented as proportions across predefined age categories (18–24, 25–34, etc.), were compared using the Chi-squared test for independence to detect significant differences in demographic interest between countries and between search terms. If necessary, Kolmogorov–Smirnov tests were employed to assess overall differences in distribution shape. Differences in click behavior (organic search result clicks, ad clicks, and no clicks) were also assessed using Chi-squared tests to examine variations by country and term. All statistical tests were two-sided, with a significance threshold set at *p* < 0.05.

## Results

### Germany

**Figure 1:**
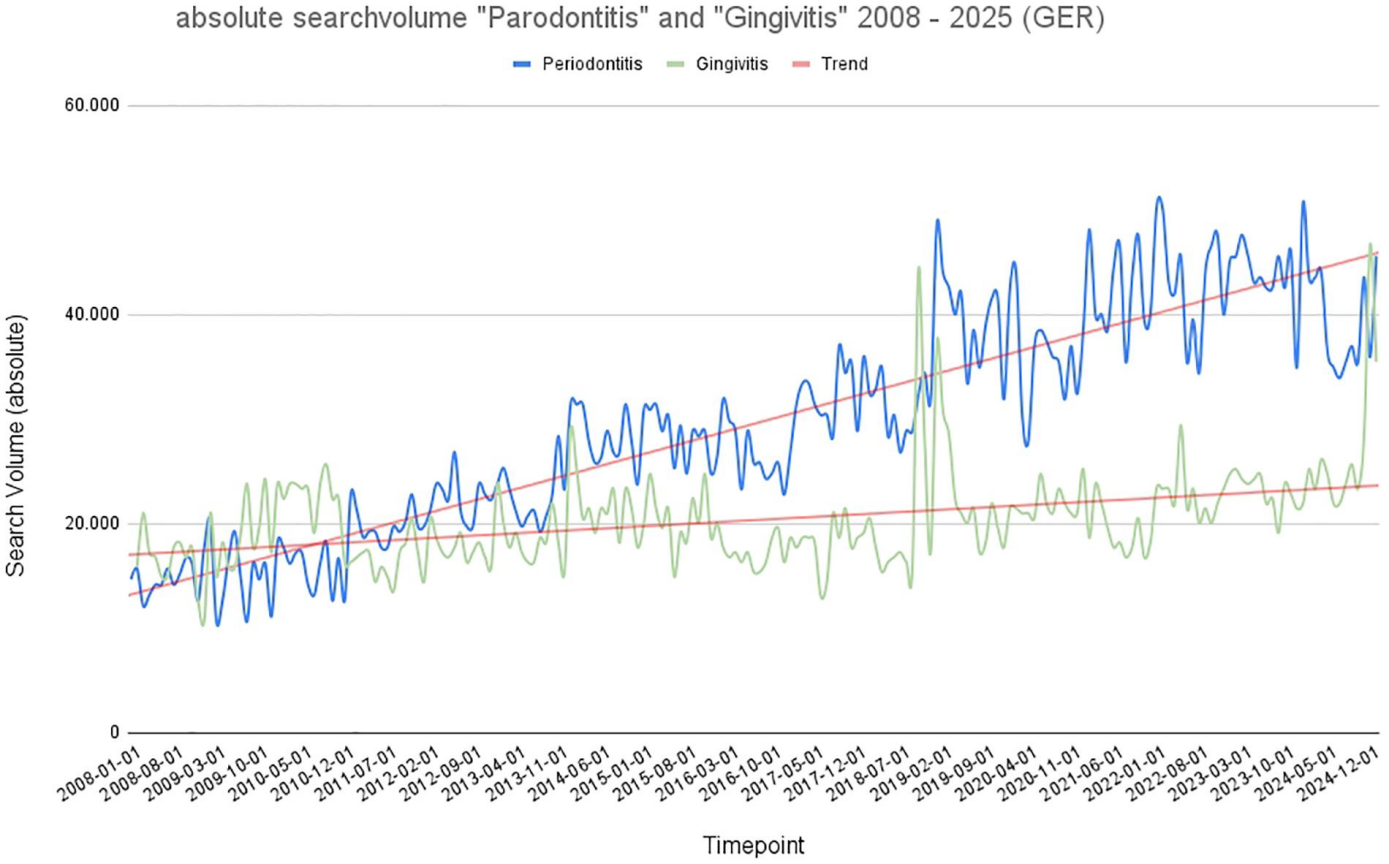
Graphical representation of absolute search volume in Germany over time (2008–2025). Blue: absolute search volume for the term “periodontitis”; green: absolute search volume for the term “gingivitis.” Red: trend line.

Our analysis shows that there was a significant (*p* < 0.001) increase in the absolute search volume for the term “periodontitis” across Germany in the last 17 years. While the monthly search volume for the search term “periodontitis” was still around 15,000 searches per month in 2008, it had risen to an average of around 50,000 searches per month by January 2025. Maximum values were reached at the beginning of 2019 and in the post COVID-19 years 2021 and 22. In the last 5 years, there was no significant increase in search queries, but fluctuated constantly around 50,000. The search volume for the search term “gingivitis” also recorded a significant (*p* < 0.001) overall increase from 18,000 monthly queries in 2008 to around 23,000 monthly queries in January 2025. Overall, a positive trend can also be seen here, but it is much more pronounced in the case of the search term “periodontitis”. There is also a clear peak for the term “gingivitis” in 2018 and 2024. What is striking for both search terms is a significant seasonal increase in search volume in January, which then falls continuously and reaches its lowest point towards the end of the year. While “gingivitis” was searched more frequently in 2008, the term “periodontitis” was searched much more frequently from 2010 onwards. This phenomenon can be seen in Figure 1 by crossing of the two graphs. Overall, in Germany, the term periodontitis is searched for significantly (*p* < 0.001) more often than gingivitis.

### Italy

**Figure 2:**
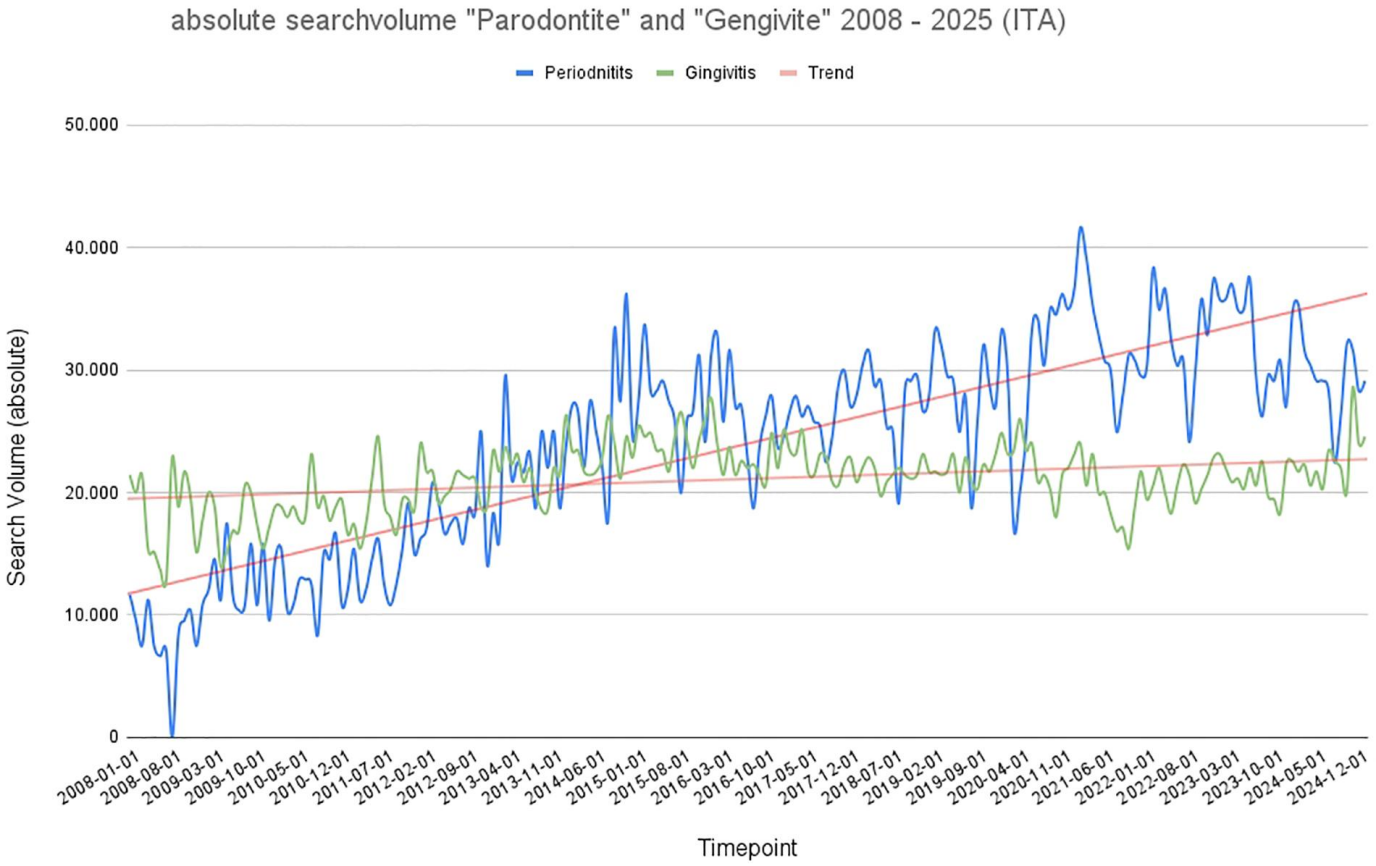
Graphical representation of absolute search volume in Italy over time (2008–2025). Blue: absolute search volume for the term “periodontitis”; green: absolute search volume for the term “gingivitis.” Red: trend line.

The search volumes show similar patterns for Italy: absolute search queries increased over time for both search terms, with the term “periodontitis” recording a significantly (*p* < 0.001) stronger increase than the term “gingivitis.” In Italy, there was also a “crossing point” in the trend lines, but this occurred in 2014, a few years later than in Germany. Overall, the search volume for gingivitis fluctuated similarly to Germany over the period under review, at around 20,000 queries per month, with a slight increase. While the absolute search volume for the term “periodontitis” was still around 12,000 searches per month in 2008, it recorded a significant (*p* < 0.001) increase to an average of 35,000 searches per month by 2025. Again, peaks in the COVID-19 years and a clear seasonality of search queries are noticeable. Notable peaks in “gingivitis” are observable in 2014 and again around 2020–2021, while “periodontitis” displayed a more uniform trajectory without significant short-term spikes (Figure 2). Italy too, shows significantly (*p* < 0.001) higher overall search volumes for periodontitis than gingivitis.

### France

**Figure 3:**
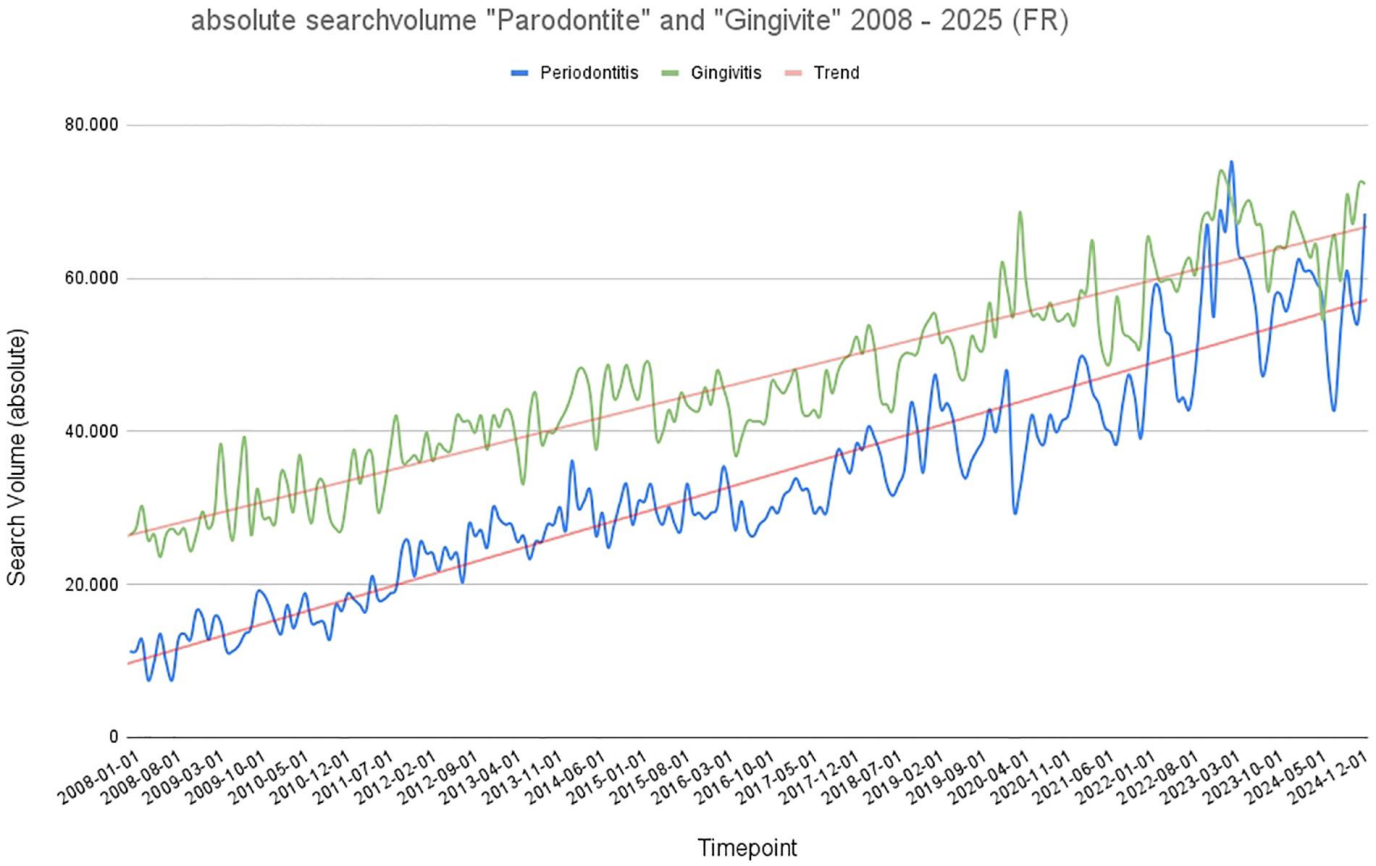
Graphical representation of absolute search volume in France over time (2008–2025). Blue: absolute search volume for the term “periodontitis”; green: absolute search volume for the term “gingivitis.” Red: trend line.

In contrast to the search histories for Germany and Italy, the graphs for the search terms “gingivitis” and “periodontitis” do not intersect in France, as illustrated in Figure 3. Although both search terms show a significant (*p* < 0.001) increase over the period under review, they run almost parallel, with the difference between the search queries becoming increasingly smaller. It is noteworthy that, in contrast to the countries described above, the absolute search volume for the term “gingivitis” consistently exceeded that of periodontitis significantly (*p* < 0.001). Gingivitis consistently registered significantly (*p* < 0.001) higher monthly search volumes than periodontitis across the period under consideration. Gingivitis ranged approximately between 25,000 and 70,000 searches per month, with the trendline suggesting continuous growth. Fluctuations were present, especially post-2018. “Periodontitis” remained between 10,000 and 30,000 with relatively smoother dynamics and a gentler increase over time. Seasonal variation appeared less pronounced in this dataset, and the divergence between the two search terms widened after 2015. A clear seasonality of search queries can also be seen in France. However, the peaks in the COVID-19 years are less pronounced than in Italy and Germany. Overall, search queries for the term gingivitis are significantly (*p* < 0.001) higher than in Italy and Germany. The absolute search volume rose from approximately 10,000 queries per month in 2008 to just under 60,000 per month in 2025 (Figure 3), exceeding the search volume in Germany and Italy.

### Spain

**Figure 4:**
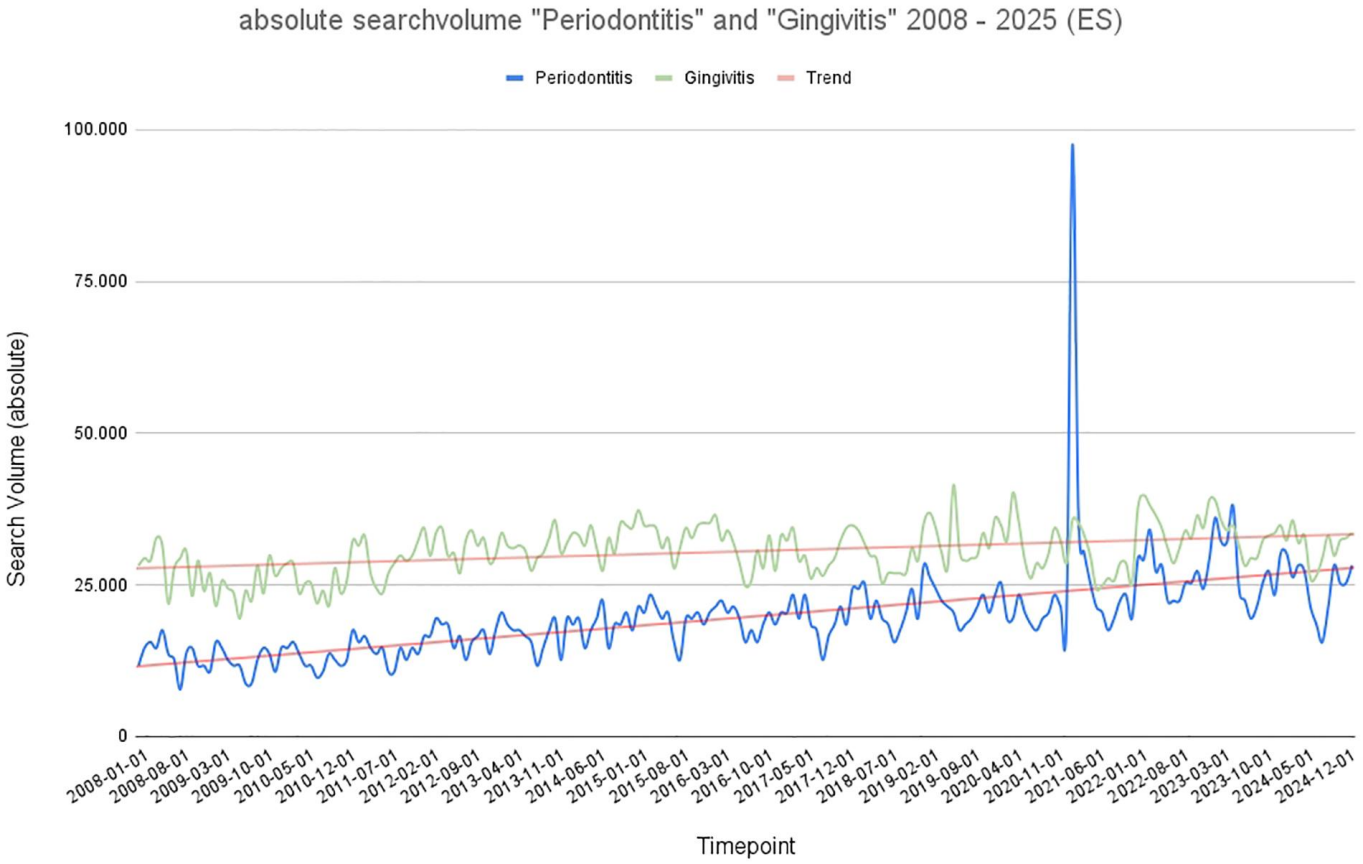
Graphical representation of absolute search volume in Spain over time (2008–2025). Blue: absolute search volume for the term “periodontitis”; green: absolute search volume for the term “gingivitis.” Red: trend line.

As shown in Figure 4 the search volume graphs for Spain are similar to those for France, but with a much flatter curve and significantly (*p* < 0.001) lower search volumes for both terms overall. The trend lines for “gingivitis” and “periodontitis” do not intersect; “gingivitis” consistently has higher search volumes within the period under review. The relatively flat increase of both curves over time and the extreme peak in 2021 are striking. “Periodontitis” and “gingivitis” showed generally lower search volumes compared to the other countries analyzed. “Periodontitis” demonstrated a gradual increase from approximately 1,000 to 5,000 monthly searches, with a relatively smooth trend line. “Gingivitis” followed a more variable course, with occasional seasonal changes in search volume, though without sustained trend disruption. The general tendency for both terms is a modest upward trend, with “periodontitis” showing slightly more consistent growth in the later years.

### USA

**Figure 5:**
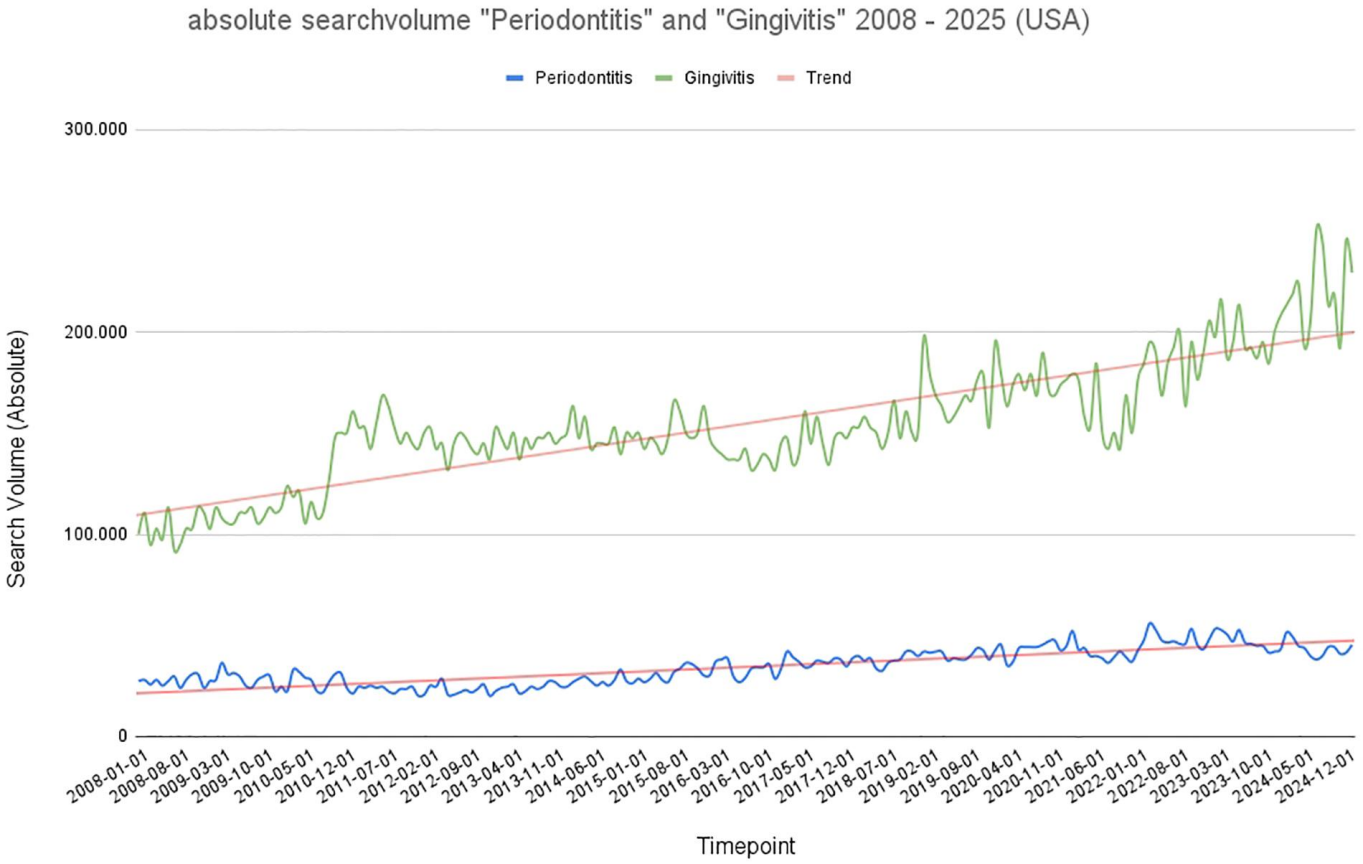
Graphical representation of absolute search volume in the USA over time (2008–2025). Blue: absolute search volume for the term “periodontitis”; green: absolute search volume for the term “gingivitis.” Red: trend line.

**Figure 6:**
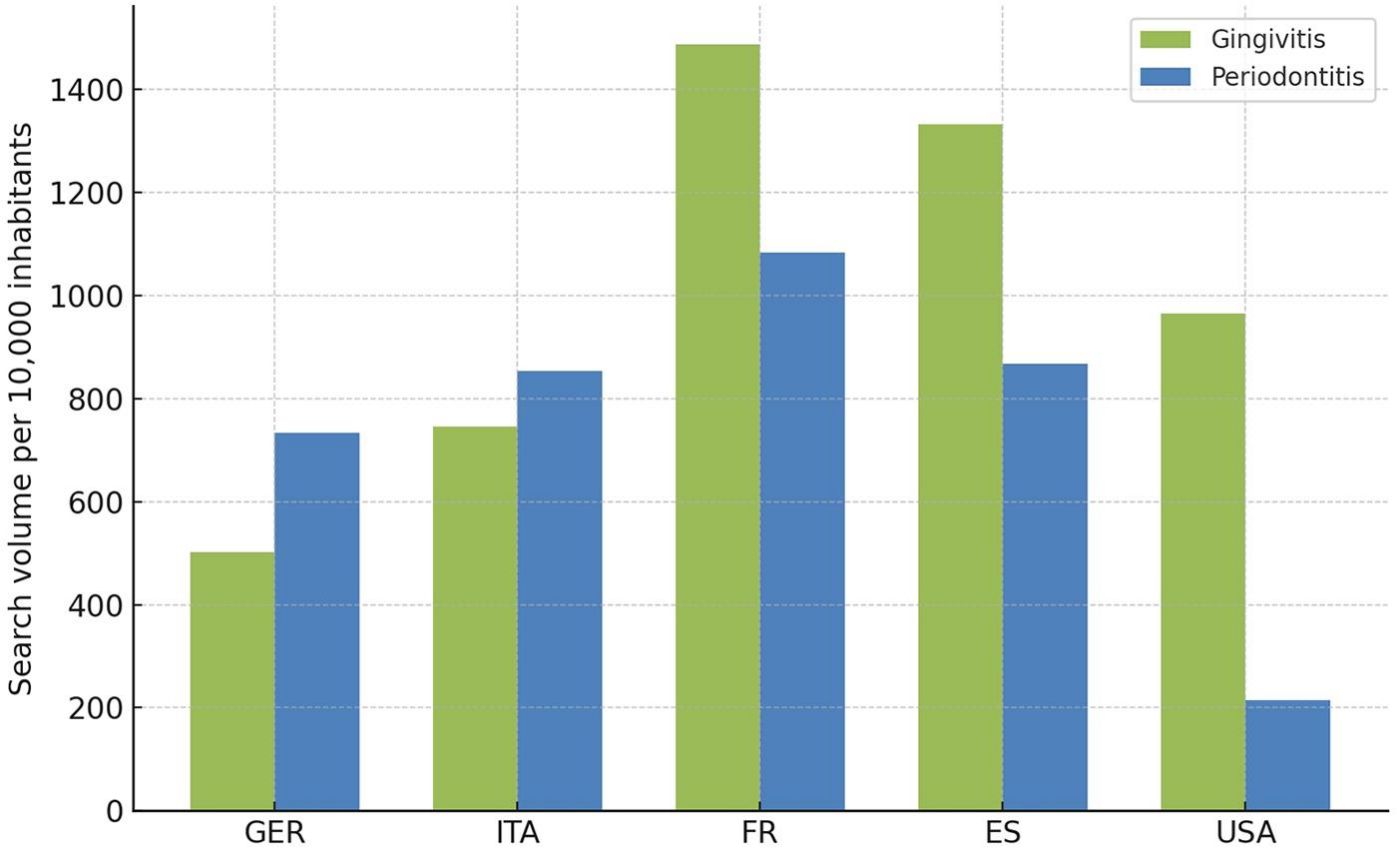
Average monthly search volume for the terms “Gingivitis” (green) and “Periodontitis” (blue) per 10,000 inhabitants in the analyzed countries (Germany, Italy, France, Spain, USA).

Also the United States demonstrated a significant (*p* < 0.001) predominance of “Gingivitis” over “Periodontitis” in public search behaviour, as shown in Figure 5. “Gingivitis” showed relatively high absolute search volumes in 2008, followed by a gradual and consistent incline through to 2025. “Parodontitis”, meanwhile, remained almost flat at a minimal level of interest throughout the entire period. In the USA, the absolute search volume for “Gingivitis” was substantially higher than that for “Parodontitis” throughout the entire time frame. “Gingivitis” searches ranged between approximately 90,000 and over 250,000 per month and displayed a consistent upward trajectory with moderate seasonal variability. A prominent discontinuity occurred around 2011–2012, after which the average volume increased. The linear trend indicates continuous growth. In contrast, “Periodontitis” searches remained significantly lower, mostly between 20,000 and 50,000, with a slight upward trend visible in the linear fit. Looking at the absolute search volume in the USA, it is noticeable that in 2008 it was only slightly higher than in Germany and other European countries. By 2025, there was only a subtle, non-significant increase in search volume, meaning that the search volumes in the USA and Germany were at a similar level in 2025. When normalized to the respective national populations, notable differences in search interest between countries became evident (Figure 6). Across the 2008–2025 period, gingivitis searches per 10,000 inhabitants were highest in France (1488.10), followed by Spain (1263.72), Italy (1226.01), Germany (501.76), and the USA (462.25). For periodontitis, France again demonstrated the highest relative search volume (1082.99), followed by Spain (1002.57), Italy (935.19), Germany (619.49), and the USA (214.75). This normalization revealed that, despite the USA exhibiting large absolute search numbers due to its population size, the relative search activity per capita was lowest among the five countries studied. Conversely, France and Spain displayed proportionally higher interest in both conditions, suggesting greater public awareness or concern regarding periodontal health in these populations.

**Figure 7:**
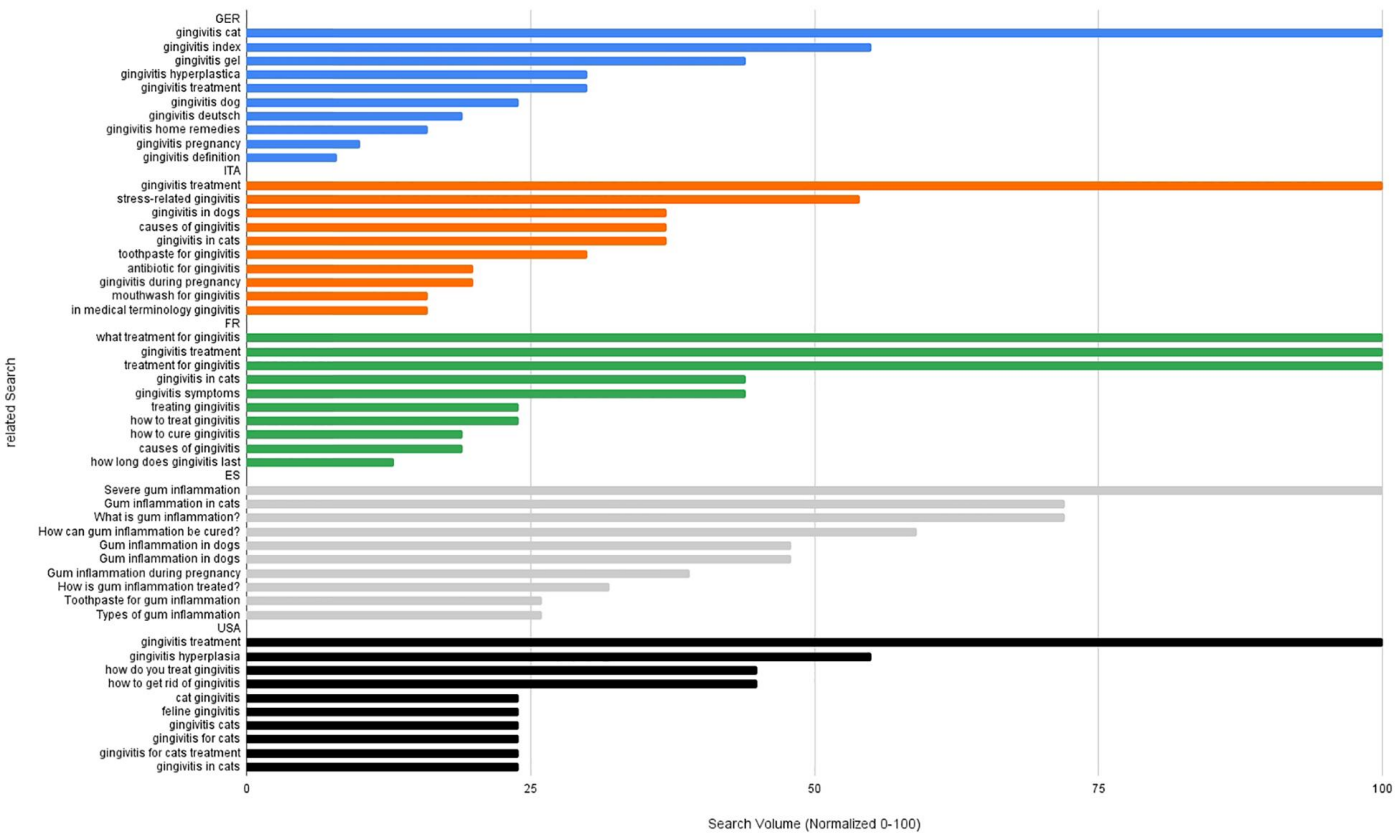
Relative search volume (normalized 0–100) of associated google queries related to gingivitis in Germany (blue), Italy (orange), France (green), Spain (grey) and in the USA (black).

**Figure 8:**
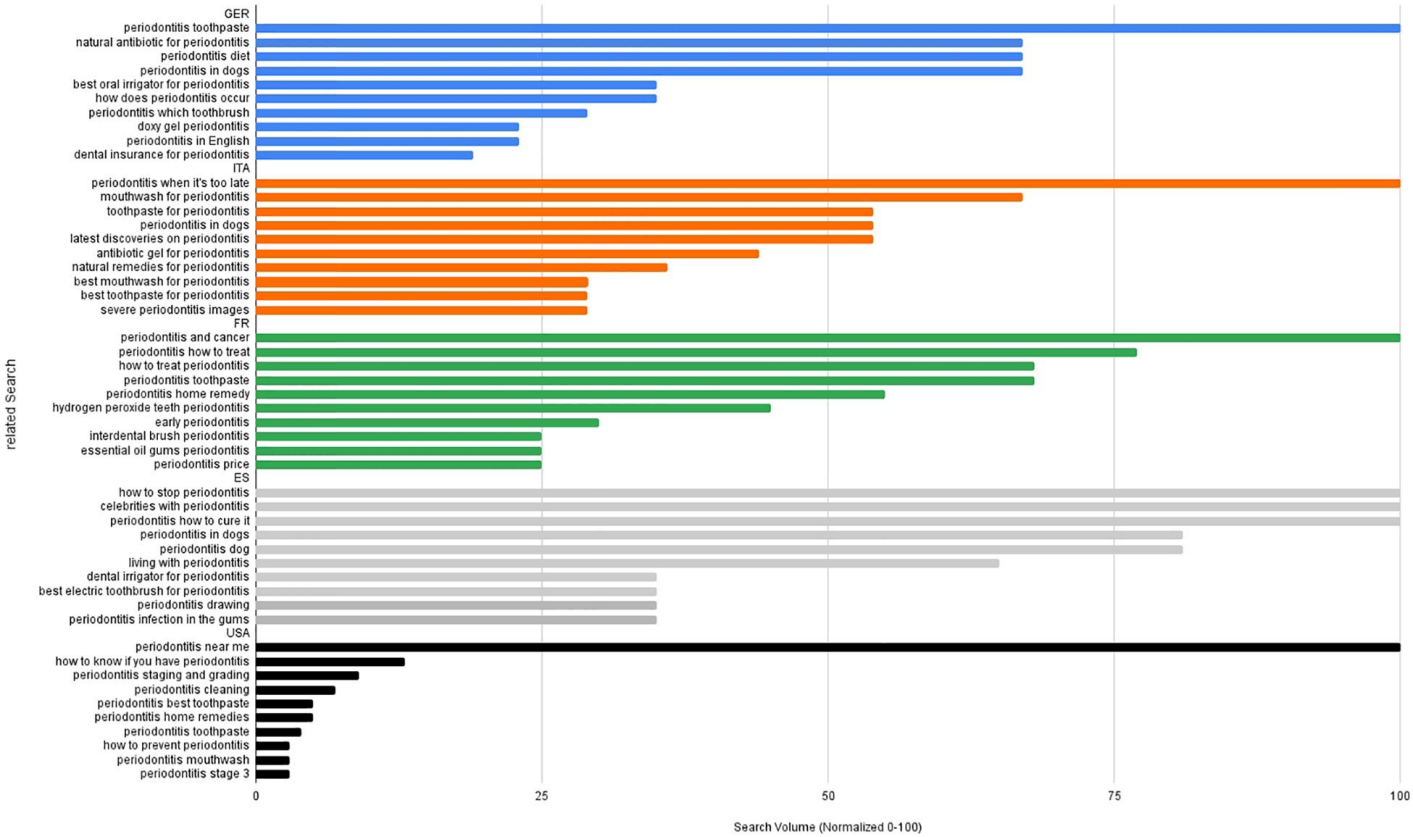
Relative search volume (normalized 0–100) of associated google queries related to periodontitis in Germany (blue), Italy (orange), France (green), Spain (grey) and in the USA (black).

An analysis of related search queries for the term “gingivitis” in Germany reveals a wide variety of interests among users, which are depicted in Figure 7. By far the most frequently searched term was “gingivitis cat” (standard value 100), indicating a high demand for information in the field of veterinary dentistry. This is followed by terms such as “gingivitis index” and “gingivitis gel”, which suggest an increased interest in diagnostic parameters and over-the-counter treatment options. Terms such as “gingivitis treatment”, “gingivitis hyperplastica”, and “gingivitis home remedies” also indicate a pronounced need for treatment options, both conventional and alternative. The frequent searches for “gingivitis dog” and “gingivitis pregnancy” also highlight the relevance of the topic across different target groups, both in a veterinary and specific human medical context (e.g., hormonal influences). Less frequent search queries such as “gingivitis definition”, “gingivitis antibiotics”, or “gingivitis pictures” indicate a more basal interest in information or for visual orientation. Overall, there is a broad spectrum of thematic relevance, which, in addition to medical information, is also strongly influenced by practical everyday and veterinary issues. An analysis of the most frequently used search queries for the term “periodontitis” is shown in Figure 8 and illustrates, that in Germany users' information needs are primarily focused on specific therapeutic measures. The most frequently searched terms were “periodontitis toothpaste”, followed by “natural antibiotic for periodontitis”, “periodontitis diet”, and “periodontitis in dogs”. These terms illustrate a strong interest in freely accessible, alternative, or supportive forms of treatment, as well as in veterinary aspects. In addition, technical aids (“best oral irrigator for periodontitis”) and pathophysiological questions (“how does periodontitis occur”) are also frequently addressed. The demand for specific products (“doxy gel”, “toothbrush”) and the search query “dental insurance for periodontitis” are also striking, reflecting an economic component in the discussion of the disease. Overall, the search trends indicate a broad interest in information that takes into account practical, preventive, and financial aspects of periodontitis.

Analysis of related search queries for “gingivitis” (Figure 7) and “periodontitis” (Figure 8) in Italy reveals clear differences in the thematic focus and information needs of users. In the area of “gingivitis”, the focus is primarily on practical and everyday aspects. The analysis of Italian search behaviour is dominated by medical and therapeutic search terms such as “gingivitis treatment”, “stress-related gingivitis”, questions about etiology (“causes of gingivitis”), and specific conditions such as “gingivitis during pregnancy”. For “periodontitis”, on the other hand, more specific and in-depth information needs can be identified. Among the Italian related search queries, the search “periodontitis when it's too late” ranks first, indicating an increased awareness of the problem. Other frequent terms relate to specific treatment measures (“mouthwash”, “toothpaste”, “antibiotic gel”) and alternative therapeutic approaches (“natural remedies”, “latest discoveries”).

The analysis of search queries relating to gingivitis (Figure 7) and periodontitis (Figure 8) in France reveals clear thematic priorities and differences in the population's information needs. Questions about treatment such as “what treatment for gingivitis”, “gingivitis treatment”, and “treatment for gingivitis” predominate, each reaching maximum search frequency. Other frequent search queries relate to symptoms, causes, and treatment in animals, especially cats. This indicates a strong practical interest in information, which is primarily focused on immediate solutions and aspects relevant to everyday life. In case of periodontitis, the greatest search interest focuses on the potential link with serious systemic diseases (“periodontitis and cancer”) and therapeutic questions (“periodontitis how to treat”, “how to treat periodontitis”). Alternative treatment approaches (“periodontitis home remedy”, “essential oil gums periodontitis”) and supportive measures such as special toothpastes or interdental brushes also play an important role. In summary, it can be observed that in France both search-terms are associated with a highly treatment-oriented search behavior. While gingivitis is primarily associated with everyday questions, periodontitis is also linked to a pronounced interest in association with systemic conditions and complementary and preventive approaches.

Analysis of Spanish search queries relating to gingivitis (Figure 7) reveals that acute and animal-related aspects are particularly prominent. The most frequently searched terms are “severe gum inflammation”, “gum inflammation in cats”, and “what is gum inflammation?” Therapeutically oriented queries such as “how can gum inflammation be cured?” and “how is gum inflammation treated?” also have a high relative search frequency. Animal contexts (“gum inflammation in dogs”) and specific life situations such as “gum inflammation during pregnancy” are also among the most frequently searched terms. In the area of periodontitis (Figure 8), search queries in Spain are particularly focused on treatment and management of the disease. The terms “how to stop periodontitis”, “celebrities with periodontitis”, and “periodontitis how to cure it” show the highest relative search intensity. Particularly striking here is the high search volume for “celebrities with periodontitis”, a term that, in comparison to the other countries examined, is unique to Spain in terms of frequency. Veterinary aspects (“periodontitis in dogs”) and everyday topics such as “living with periodontitis” or specific aids (“dental irrigator for periodontitis”, “best electric toothbrush for periodontitis”) are also strongly represented.

In the US, searches for gingivitis (Figure 7) show a strong interest in specific treatment measures. The term “gingivitis treatment” has by far the highest search volume, followed by “gingivitis hyperplasia” and general questions about treatment such as “how do you treat gingivitis” and “how to get rid of gingivitis”. There is also a noticeable presence of veterinary terms, particularly in connection with gingivitis in cats (“cat gingivitis”, “feline gingiviti”, “gingivitis in cats”), which suggests a broad public awareness of oral diseases in pets. When it comes to periodontitis (Figure 8), the search query “periodontitis near me” clearly dominates all others, indicating a strong practical need for information about local treatment options. Other relevant search terms relate to self-assessment (“how to know if you have periodontitis”) and diagnostic aspects (“periodontitis staging and grading”) and home care (“periodontitis cleaning”, “periodontitis best toothpaste”, “home remedies”). The search patterns indicate a highly individualized and action-oriented interest in the US context, with both professional and self-initiated measures being at the forefront.

The results can be further analyzed using the “Ubersuggest” software. With the help of this program, it is possible to track the percentage of users who clicked on paid advertising, SEO ads, or no displayed results (Table 1). Furthermore, the age groups of users and the type of device (mobile vs. desktop) can be analyzed. The analysis is limited to the entire last year within the program.

**Table 1:** User click behavior on google search results for “gingivitis” and “periodontitis” across five countries. Table displays the absolute number of clicks on search results (Search), advertisement clicks (Ads), and non-click interactions (No clicks) for both search terms in Germany, Italy, France, Spain, and the USA. It also includes the relative ratio of gingivitis to periodontitis search volume [Gingivitis vs. Periodontitis (%)].

Country	Search (periodontitis)	Ads (periodontitis)	No clicks (periodontitis)	Search (gingivitis)	Ads (gingivitis)	No clicks (gingivitis)	Gingivitis vs. periodontitis (%)
GER	11,792	4,729	16,580	6,607	2,263	9,231	56.0%
ITA	13,629	4,956	14,514	5,638	758	5,704	41.4%
FR	21,310	5,450	22,740	17,067	4,567	18,866	80.1%
ES	9,951	1,555	15,594	10,311	1,975	14,814	103.6%
USA	15,690	1,093	57,217	57,754	3,030	185,216	368.1%

The analysis of click behavior in relation to the search terms “gingivitis” and “periodontitis” shows clear differences both between the two diseases and between countries. Overall, the majority of search queries in all countries do not result in active click behavior, which indicates a potentially low interaction rate with search results or a merely exploratory search for information. For periodontitis, the proportion of users who do not click on any search results is significantly high in all countries. This is particularly pronounced in the US, where 72% of users (57,217 out of 74,000) do not engage in any further interaction after their search. In Spain (56.3%), Italy (47.9%), and Germany (43.3%), the proportion of “no clicks” is also significantly higher than the proportion of active clicks. France is an exception here: with 22,740 no-click searches out of 49,500 total interactions, the proportion is slightly lower (46%), which suggests a higher level of active engagement with search results compared to other countries. The ratio between organic clicks (on unpaid search results) and paid ads also varies between countries. Germany and Italy, for example, have relatively high proportions of ad clicks, with 4,729 and 4,956 clicks on ads respectively (Germany: 20.1%; Italy: 26.7%), which indicates a certain effectiveness of paid advertising in these markets. In the US, on the other hand, this share is only 6.5%, even though the absolute search volume is highest there overall. This suggests that search behavior in the US is predominantly organic. In the case of gingivitis, the differences in click behavior are even more pronounced. In the US, an overwhelming majority of users (185,216 out of 245,000) do not click on any of the search results, which corresponds to a share of over 75%. This pattern is also evident in France (47.4%) and Spain (48.3%). Germany, on the other hand, stands out with a higher proportion of clicks on organic results (6,607 out of 18,101 total interactions; approx. 36.5%). The proportion of ad clicks remains relatively low for gingivitis in all countries, with the exception of France (4,567 clicks on ads, 24.2%) and Germany (2,263 clicks, 12.5%). Overall, it can be said that for both search terms, a high proportion of users do not actively click, which could indicate passive information gathering or the use of snippets and preview texts on the results page. The proportion of paid clicks is higher for periodontitis than for gingivitis in several countries, suggesting that commercial providers advertise more specifically in the field of periodontal therapy or that users assume a greater urgency for treatment in this area. The US consistently shows the lowest advertising interaction, even though it has the highest absolute search volume—this may indicate high organic content quality or advertising fatigue in the user experience. Analysis of the percentage ratio between total search queries for gingivitis and periodontitis shows significant country-specific differences. In the US, interest in gingivitis clearly dominates, accounting for around 3,3-fold (332%) of searches compared to periodontitis. This indicates a strong preventive search behavior, which may be influenced by higher public health awareness or different dental care systems. In Spain, the ratio is balanced (100%), suggesting that both diseases are perceived as equally important. France shows a slight shift toward periodontitis with approximately 82%, while in Germany (55%) and Italy (37%), interest is significantly more focused on periodontitis. In summary, the data clearly show that both click behavior and the effectiveness of advertising vary significantly between countries and disease terms.

The analysis of the age distribution of search queries for the terms “gingivitis” (Table 2) and “periodontitis” (Table 3) reveals clear differences both between the two conditions and between the countries examined. Overall, search queries for gingivitis tend to come from younger user groups, while periodontitis is more frequently researched by middle-aged individuals. This distribution is closely related to the clinical epidemiology of both diseases. In the case of gingivitis, the 18–34 age group dominates in almost all countries. Germany is particularly striking, with 64.7% of search queries originating from this age cohort (15.3% aged 18–24 and 49.4% aged 25–34). Italy has a similarly high proportion, with 36.9% in both groups (73.8% in total), followed by France with a total of 56.4%. In contrast, periodontitis search queries are more concentrated in the middle age groups. In France, the proportion of 35- to 44-year-olds is 40.9%, in Germany 39.4%, followed by 25- to 34-year-olds in both countries. These two age groups are also dominant in Italy (37.0% and 36.6%). This distribution reflects the natural course of the disease, as periodontitis usually only becomes clinically noticable by patients from the third to fourth decade of life. Thus, a clear shift in the search for information on periodontitis can be observed in older population groups, who are often already affected. A comparison between countries also reveals differences in digital usage. The US has a relatively high proportion of 18- to 24-year-old users for both gingivitis (31.2%) and periodontitis (29.3%). Spain shows a relatively even distribution across the 18–44 age group, with a total share of over 90% for both search terms. It is striking that in all countries, the age groups under 18 and over 65 are almost irrelevant in the data. No significant differences were found in the age distribution of search interest between the terms *periodontitis* and *gingivitis* within any of the five countries analyzed. Chi-squared tests revealed non-significant results in all cases (all *p* > 0.09), indicating similar age-related search patterns for both terms across the 18–64 age groups.

**Table 2:** Age distribution of users searching for “gingivitis” across five countries (in %).

Country	0–18y	18–24y	25–34y	35–44y	45–54y	55–64y	65+ y
GER	0%	15.3%	49.4%	29.4%	3.5%	2.4%	0%
ITA	0%	36.9%	36.9%	22.9%	3.2%	0.0%	0%
FR	0%	20.4%	36.0%	36.0%	4.9%	2.7%	0%
ES	0%	27.2%	36.4%	27.2%	6.7%	2.5%	0%
USA	0%	31.2%	9.0%	41.5%	9.4%	8.9%	0%

**Table 3:** Age distribution of users searching for “periodontitis” across five countries (in %).

Country	0–18y	18–24y	25–34y	35–44y	45–54y	55–64y	65+ y
GER	0%	17.7%	36.0%	39.4%	4.2%	2.5%	0%
ITA	0%	23.5%	37.0%	36.6%	1.7%	1.2%	0%
FR	0%	15.7%	26.1%	40.9%	6.5%	10.7%	0%
ES	0%	28.4%	34.3%	27.8%	5.7%	3.9%	0%
USA	0%	29.3%	9.6%	37.6%	11.1%	12.4%	0%

In summary, it appears that digital demand for information on gingivitis and periodontitis exhibits age- and country-specific patterns. While gingivitis is predominantly an issue for younger adults, interest in periodontitis is more pronounced among middle-aged adults. These findings are particularly important for targeted prevention strategies and digital health communication, as they suggest that a different approach is needed for different age groups. Analyzing device-related search volumes for the terms “periodontitis” and “gingivitis” (Table 4) in five countries—Germany, Italy, France, Spain, and the US—reveals clear differences in user behavior with regard to preferred devices. In all countries surveyed, mobile searches dominate over desktop devices. This result is in line with the global trend of increasingly obtaining health-related information via smartphones or tablets. In Germany, the share of mobile searches for “periodontitis” is 60.9%, while it is significantly higher for “gingivitis” at 76.0%. Italy has the highest mobile search share in a country comparison, with 78.6% for “periodontitis” and 76.8% for “gingivitis.” This result indicates a particularly strong mobile-oriented health information behavior. France stands out with a relatively balanced distribution of access: 54.7% of searches for “periodontitis” and 54.6% for “gingivitis” are made via mobile devices. This balance suggests that different types of devices are used relatively evenly in France. Spain shows above-average mobile usage, with 62.0% of searches for “periodontitis” and 60.0% for “gingivitis” coming from mobile devices, but still lagging behind Italy and the US. Finally, in the US, the mobile share is 70.1% for “periodontitis” and 66.9% for “gingivitis,” underscoring the strong preference of the US population for mobile devices as a source of information. A comparative look at the two search terms shows that in almost all countries, the mobile share is higher for “gingivitis” than for “periodontitis”. In a transcontinental comparison, the US has consistently higher mobile usage rates than most European countries, at 70.1% (periodontitis) and 66.9% (gingivitis). In Europe, the mobile shares vary more widely.

**Table 4:** Table presents absolute search volumes for mobile and desktop devices related to “gingivitis” and “periodontitis” in Germany, Italy, France, Spain, and the USA, along with the corresponding mobile-to-desktop share percentages.

Country	Periodontitis mobile	Desktop	% Mobile	Gingivitis mobile	Desktop	% Mobile
GER	20,145	12,955	60.9%	11,245	3,555	76.0%
ITA	26,015	7,085	78.6%	9,295	2,805	76.8%
FR	27,065	22,435	54.7%	22,133	18,367	54.6%
ES	16,816	10,284	62.0%	16,260	10,840	60.0%
USA	51,847	22,153	70.1%	201,557	99,443	66.9%

## Discussion

This study offers a comprehensive analysis of digital search behavior related to periodontal diseases across five countries over a 17-year period. While a small number of info-epidemiological studies have used search term analysis data in dentistry, these have mainly focused on other oral health conditions such as oral cancer or dental pain. To the best of our knowledge, gingivitis and periodontitis have not yet been systematically examined using long-term, comparative search engine analytics. By addressing these highly prevalent periodontal diseases across five countries over 17 years, the present study extends the scope of digital oral health research and provides novel insights into public information-seeking behavior. The increasing trend in search volumes for both “gingivitis” and “periodontitis” reflects a rising public interest and demand for oral health and highlights the relevance of digital media as an essential source of health information. Notably, country-specific patterns, demographic differences, and device usage provide valuable insights into the nature and context of health information-seeking behavior.

From a chronological perspective, all countries demonstrate a positive trend in search volumes, albeit with marked differences in magnitude and focus. The general increase in search volume for “periodontitis” and “gingivitis” over time can be partly explained by the continuously rising incidence of the diseases (9, 10, 25, 26). In Germany and Italy, “periodontitis” overtook “gingivitis” in relative and absolute search volumes over the past decade, with a clear crossing point around 2010 and 2014, respectively. This shift may indicate an increased public awareness of the chronic and irreversible consequences associated with periodontitis, particularly in light of its reclassification in the 2017 EFP/AAP consensus and its subsequent integration into national insurance frameworks (27). Seasonal patterns, especially consistent peaks in January, suggest a cyclical motivation for health-related searches, possibly linked to annual resolutions or preventive health campaigns. The recurrent January peaks observed across countries may reflect increased health awareness linked to New Year's resolutions, delayed care-seeking following holiday periods, or the beginning of new insurance or reimbursement cycles. These explanations remain hypothetical but are consistent with seasonal patterns observed in other digital health studies. In contrast, France and Spain show a persistent predominance of “gingivitis” in the search data, with France exhibiting the highest absolute search volumes for this term across the entire period. This could reflect a public focus on early-stage or reversible gum inflammation, perhaps influenced by greater emphasis on prevention or cosmetic oral health concerns. The United States present a unique case: despite high absolute search volumes overall, interest in “gingivitis” remain substantially higher than in “periodontitis”, with a gingivitis-to-periodontitis ratio of over 3:1. This finding aligns with a more preventive search behavior and may be shaped by differences in health system structure, public campaigns, or dental service access. Click behavior further emphasizes the heterogeneity of search engagement. Across all countries, a large proportion of users (often exceeding 50%) did not click on any of the results, pointing to a preference for consuming information directly from search engine previews or featured snippets. Germany and France show relatively higher engagement with organic results, whereas the United States demonstrated minimal interaction with paid advertisements despite the highest search volume—suggesting either a high trust in organic content or a saturation of ad exposure. Interestingly, advertising click-through rates are significantly higher (p < 0.001) in Italy and France, which may imply a greater receptivity to promoted content in these populations. Cross-national differences in search behavior may also be influenced by socioeconomic factors and healthcare system characteristics. Variations in insurance coverage, access to preventive dental care, public awareness campaigns, and cost structures may shape how and when individuals seek information online. In this context, the introduction of the new periodontal classification in 2017 may have contributed to increased visibility and awareness of periodontitis, as it was accompanied in some countries—such as Germany—by intensified professional and public education efforts from periodontal societies. In addition, the implementation of revised national reimbursement guidelines for periodontal treatment, including the recent German statutory health insurance directive, may have further stimulated public interest and information-seeking behavior. At the same time, socioeconomic factors must be considered, as unequal access to the internet or digital devices may limit participation in online health information seeking for certain population groups. Age-stratified data reveal that information-seeking on “gingivitis” is concentrated among younger adults (18–34 years), particularly in Germany (64.7%) and Italy (73.8%). This is consistent with the early onset and reversibility of gingival inflammation, which tends to affect adolescents and young adults and may trigger proactive searching for home remedies or oral hygiene strategies. In contrast, searches for “periodontitis” are most prominent among users aged 25–44 years, corresponding with the typical clinical manifestation of periodontal destruction. Notably, users above 65 years are underrepresented in all datasets, likely due to digital exclusion. However, this is expected to shift with ongoing demographic changes and increasing digital literacy among aging populations. Device usage analysis reveals a clear global trend toward mobile dominance. Mobile searches account for over 60% of queries in nearly all countries, with the highest rates observed in Italy (up to 78.6%) and the United States. This underscores the necessity for mobile-optimized educational content, particularly for younger and mid-aged user groups who predominantly access health information via smartphones. France is the only country to demonstrate an almost equal distribution between mobile and desktop devices, indicating a more heterogeneous media consumption pattern. The analysis of associated search terms highlights additional thematic divergences. In Germany and Italy, related queries are heavily centered on treatment modalities, such as toothpastes, antibiotic gels, and oral irrigators, reflecting a pragmatic interest in accessible and problem orientated solutions. Spain shows a unique behavioral pattern with a high search frequency for “celebrities with periodontitis,” suggesting a psychosocial or identity-related component in disease perception. The high search frequency for “celebrities with periodontitis” in Spain may reflect culturally specific drivers of health awareness, including strong media engagement with public figures and the role of celebrities in shaping health-related narratives. Such mechanisms may amplify public attention to diseases through identification and social relevance. Across all countries, there is a substantial interest in veterinary aspects, particularly concerning “gingivitis in cats”, which points to a trans-species concern for oral health. These findings provide important implications for clinical practice and public health strategies. The high search volumes and their consistent increase across countries support the integration of digital behavior analytics into periodontal awareness and prevention programs. Dental associations and professional societies could use search behavior insights to tailor digital awareness campaigns, optimize search-engine–visible educational content, and address common patient concerns. The high proportion of “no-click” searches may further indicate unmet informational needs or reliance on search-result previews, underscoring the importance of clear, accessible, and trustworthy online content. Tailored online content, optimized for mobile access and targeting specific age demographics, can improve digital health literacy and foster earlier intervention. Additionally, understanding national differences in click behavior and engagement can guide the design of more effective search engine optimization and advertising strategies in the healthcare sector.

Nevertheless, limitations must be acknowledged. Search engine data do not provide direct clinical correlations, and the identity or intention of the searcher (e.g., patient, student, caregiver) remains unknown. Another important limitation relates to the selection of search terms. The analysis was deliberately restricted to the defined keywords “gingivitis” and “periodontitis” and their language-specific translations in order to ensure methodological consistency and cross-country comparability. However, this approach does not fully capture the breadth of public search behavior, which often includes lay terminology, symptom-based expressions (e.g., “gum disease”, “bleeding gums”, “loose teeth”), spelling variations, or complex multi-word queries. Future studies should expand this approach by systematically integrating plain language terms and symptom-oriented queries to further refine the understanding of population-level information needs*.* Furthermore a relevant limitation is the substantial presence of veterinary-related queries, which may inflate search volumes intended to reflect human periodontal health interest, especially in Germany and the United States. Their influence should be considered when interpreting search volumes. Although validated translations were used, cultural and linguistic differences in symptom perception, terminology, and health communication may influence search behavior beyond literal translation accuracy. Search engine data may reflect public interest rather than actual disease prevalence and therefore should be interpreted with care. In addition, results are influenced by Google's proprietary algorithms and by third-party demographic estimates (e.g., from Ubersuggest), which may not be fully representative and cannot be independently validated. This analysis is based on data provided by Google Trends, Glimpse, and Ubersuggest, which compile large-scale Google search queries using proprietary processing workflows. Although absolute search volumes are reported, the exact mechanisms by which these platforms aggregate, normalize, and classify queries are not fully transparent. Therefore, while the data allow robust comparative and longitudinal analyses, some uncertainty remains regarding the underlying categorization and filtering procedures. Moreover, the analysis is restricted to Google-based platforms, although their market share justifies this focus in the studied countries (24). Given the growing integration of artificial intelligence into search platforms—most notably through tools such as ChatGPT or Google's Search Generative Experience (SGE)—future analyses of search behavior should also consider data from these AI-driven interfaces. As users increasingly turn to conversational agents for health-related information, search dynamics may shift away from traditional keyword-based queries toward more complex, dialogue-based interactions. This evolution in user behavior may affect both the volume and the nature of queries related to periodontal diseases and should be taken into account in future surveillance strategies. The high search frequency for periodontal disease in animals, particularly in veterinary contexts, should also be acknowledged, as it may introduce a bias into the interpretation of search volumes intended to reflect human health interest. Based on these findings, future research may test hypotheses such as whether targeted digital campaigns alter search behavior, whether changes in reimbursement policies influence public awareness,.

In conclusion, this study demonstrates that digital search behavior, offers valuable insights into public interest and unmet informational needs related to periodontal diseases. Cross-national and demographic differences emphasize the necessity of tailored, age-appropriate, and mobile-optimized communication strategies. The strong demand for treatment-related content highlights the importance of accessible, trustworthy education to support individuals in managing their oral health. Promoting early awareness—especially through targeted digital campaigns—not only supports patient well-being and quality of life by enabling timely prevention and treatment but also contributes to reducing the long-term societal and economic burden associated with advanced periodontal disease. These findings highlight also that search engine analytics can serve as a valuable tool for identifying patients' needs and interests—supporting the principles of personalized medicine. Strengthening digital health literacy in this context serves both individual and public health interests.

## Data Availability

The raw data supporting the conclusions of this article will be made available by the authors, without undue reservation.
